# Effervescent cannabidiol solid dispersion-doped dissolving microneedles for boosted melanoma therapy via the “TRPV1-NFATc1-ATF3” pathway and tumor microenvironment engineering

**DOI:** 10.1186/s40824-023-00390-x

**Published:** 2023-05-18

**Authors:** Jiachen Shi, Qiuling Ma, Wenting Su, Congyan Liu, Huangqin Zhang, Yuping Liu, Xiaoqi Li, Xi Jiang, Chang Ge, Fei Kong, Yan Chen, Ding Qu

**Affiliations:** 1 Affiliated Hospital of Integrated Traditional Chinese and Western Medicine, Nanjing University of Chinese Medicine, Nanjing, 210028 China; 2 Jiangsu Province Academy of Traditional Chinese Medicine, Nanjing, 210028 China

**Keywords:** Dissolving microneedles, Melanoma, Cannabidiol, Effervescent, Tumor microenvironment, Ca^2+^ influx

## Abstract

**Background:**

Conventional dissolving microneedles (DMNs) face significant challenges in anti-melanoma therapy due to the lack of active thrust to achieve efficient transdermal drug delivery and intra-tumoral penetration.

**Methods:**

In this study, the effervescent cannabidiol solid dispersion-doped dissolving microneedles (Ef/CBD-SD@DMNs) composed of the combined effervescent components (CaCO_3_ & NaHCO_3_) and CBD-based solid dispersion (CBD-SD) were facilely fabricated by the “one-step micro-molding” method for boosted transdermal and tumoral delivery of cannabidiol (CBD).

**Results:**

Upon pressing into the skin, Ef/CBD-SD@DMNs rapidly produce CO_2_ bubbles through proton elimination, significantly enhancing the skin permeation and tumoral penetration of CBD. Once reaching the tumors, Ef/CBD-SD@DMNs can activate transient receptor potential vanilloid 1 (TRPV1) to increase Ca^2+^ influx and inhibit the downstream NFATc1-ATF3 signal to induce cell apoptosis. Additionally, Ef/CBD-SD@DMNs raise intra-tumoral pH environment to trigger the engineering of the tumor microenvironment (TME), including the M1 polarization of tumor-associated macrophages (TAMs) and increase of T cells infiltration. The introduction of Ca^2+^ can not only amplify the effervescent effect but also provide sufficient Ca^2+^ with CBD to potentiate the anti-melanoma efficacy. Such a “one stone, two birds” strategy combines the advantages of effervescent effects on transdermal delivery and TME regulation, creating favorable therapeutic conditions for CBD to obtain stronger inhibition of melanoma growth in vitro and in vivo.

**Conclusions:**

This study holds promising potential in the transdermal delivery of CBD for melanoma therapy and offers a facile tool for transdermal therapies of skin tumors.

**Supplementary Information:**

The online version contains supplementary material available at 10.1186/s40824-023-00390-x.

## Background

Melanoma, a rare skin cancer originating from melanocytes, accounts for only approximately 1% of all skin tumors but is considered the most lethal due to its high invasiveness [1]. Chemotherapy remains the most significant adjuvant therapy following surgical resection; however, its effectiveness in treating melanoma patients is controversial due to severe systemic toxicity and poor tumor targeting [2]. Transient receptor potential vanilloid (TRPV), an ion channel protein involved in regulating intracellular calcium ion concentration, plays a crucial role in melanoma. Studies have shown that the activation of TRPV can affect survival and proliferation of melanoma cells (A2058 and A375) by altering the intracellular and extracellular Ca^2+^ concentration [3–5], making it one of the most promising targets for melanoma therapy [6]. Cannabidiol, as a TRPV1 agonist, can activate the TRPV1 channel by changing the conformation of the channel protein, leading to the opening of the channel pore and allowing the influx of Ca^2+^[7]. Previous studies have demonstrated that the influx of Ca^2+^ triggers the “Ca^2+^ influx-NFATc1-ATF3” downstream signaling pathway that involved in the melanoma development [3]. However, the correlation between CBD and “Ca^2+^ influx-NFATc1-ATF3” pathways has not been established. Furthermore, CBD is an extremely water-insoluble compound with some non-ignorable drawbacks, such as low oral bioavailability and poor thermal stability [8]. Therefore, designing a suitable CBD-associated formulation for the treatment of melanoma is highly desired from the perspective of drug delivery.

The stratum corneum, which is the outermost layer of human skin, consists of 15 ~ 20 layers of fat-depleted and protein-rich keratinocytes that serve as the primary barrier to protect the skin from foreign substances [3]. Microneedle-mediated transdermal drug delivery can overcome the obstacles presented by the stratum corneum, delivering drugs directly to topical lesions and thereby improving transdermal delivery efficiency [9–11]. This makes microneedles a promising tool for delivering active ingredients in the treatment of melanoma [12–14]. Microneedle delivery systems can be classified according to their matrices and preparation technologies, including dissolving microneedles (DMNs)[15], coated microneedles [16], hollow microneedles [17], solid microneedles [18], and others. DMNs are the self-absorbed device that no further actions are needed after the administration, gradually becoming the preferred type for microneedle designs [19]. However, DMNs face certain challenges in directly loading hydrophobic CBD because their matrix is hydrophilic [20]. Assembling CBD as a water-soluble module in advance is a potential approach to fabricating CBD-loaded DMNs. With the rapid development of materials science, there are now various mature technologies available for hydrophilizing CBD, such as solid dispersions [21], microemulsions [22], liposomes [23], electrospun fibers [24], and more. Polyvinyl pyrrolidone (PVP) has been employed in both the preparation of solid dispersions [25–27] and DMNs [28, 29], indicating a potential matrix candidate for assembling CBD-associated DMNs.

Effective transdermal drug delivery relies on deep penetration into the skin. Effervescent compounds, which produce gas under specific conditions, might be a solution for achieving deep penetration. The gas bubbles generated by effervescent components create temporary pores or channels through which drugs can diffuse into the deeper layers of the skin. Miguel and coworkers [30] reported on a strategy of active penetration-boosting by producing hydrogen bubbles inside the tumor tissues based on the reaction between magnesium particles and intra-tumoral protons. Likewise, Ke and coworkers [31] designed a microneedle system that incorporates sodium bicarbonate (NaHCO_3_) and rapidly forms CO_2_ bubbles to separate the adhesive backing and drug-loaded arrays after skin insertion. These studies demonstrate that such bubble-produced strategies are capable of actively pushing the drug deep permeation, highlighting the promising prospects in topical drug delivery. Melanoma is characterized by a mildly acidic microenvironment, which not only creates favorable conditions for effervescent compounds to produce gas but also contributes to the deterioration of the tumor microenvironment (TME) by reducing the viability and proliferation of immune cells [32–34]. In addition, some effervescent components, such as NaHCO_3_ and CaCO_3_, can act as intra-tumoral pH regulators by neutralizing excess protons and increasing the concentration of bicarbonate and bicarbonate ions [35–37], resulting in the M1 repolarization of tumor-associated macrophages (TAMs)[38] and activating the entire TME [39, 40]. Therefore, effervescent components are potentially suitable for the design of multi-functional DMNs for the comprehensive treatment of melanoma.

**Scheme 1:**
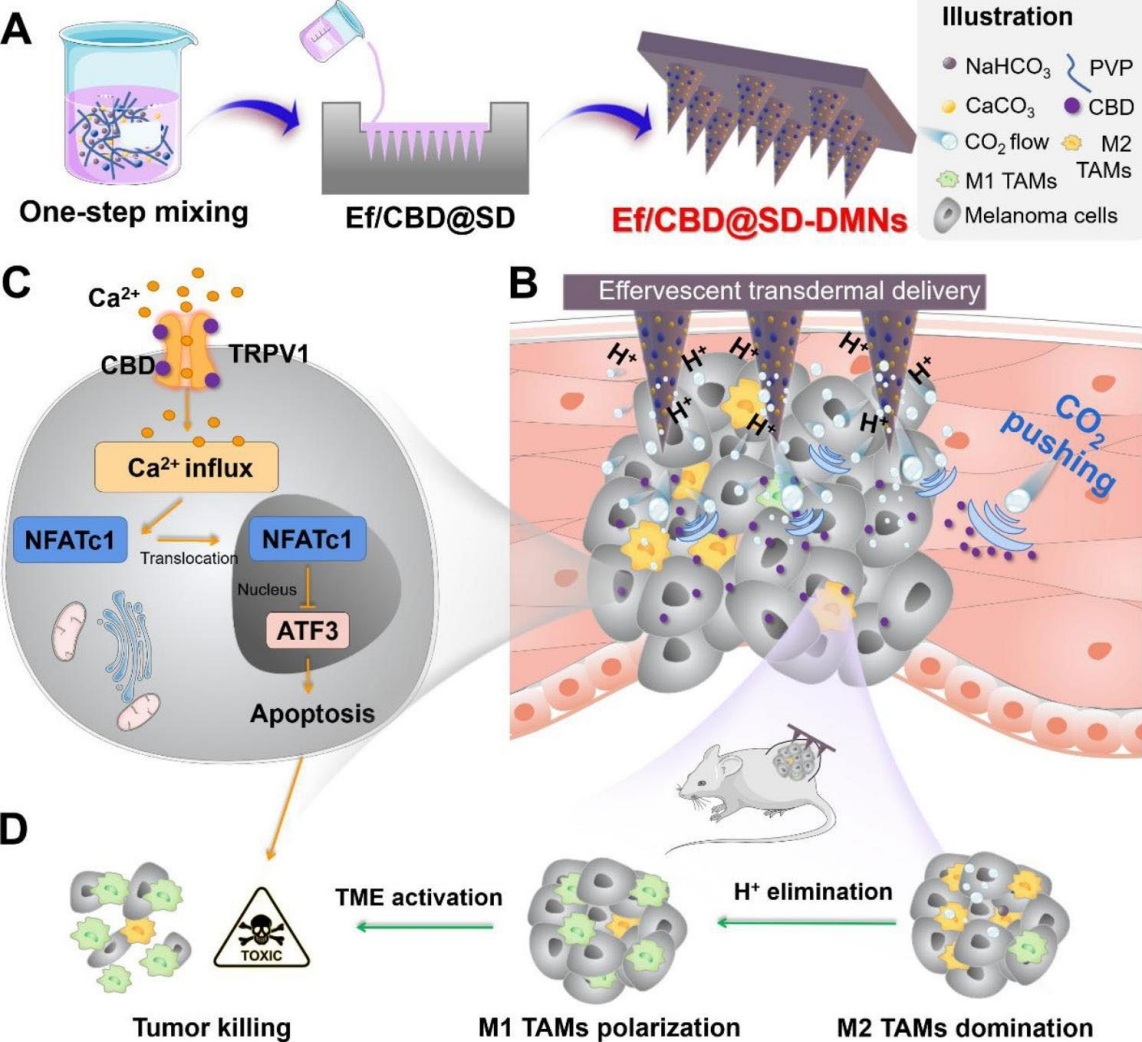
Schematic illustration of fabrication, active transdermal delivery, and synergistic antitumor mechanism of Ef/CBD-SD@DMNs. The CO_2_ bubbles generated by the combined effervescent components (CaCO_3_ & NaHCO_3_) push CBD deep penetration, consequently improving transdermal and tumoral delivery. The Ca^2+^ influx on one hand triggers the “TRPV1-NFATc1-ATF3” pathway for melanoma apoptosis and on the other hand results in the engineering of the tumor microenvironment, collaboratively boosting anti-melanoma therapy

Herein, we developed CBD solid dispersion-based effervescent DMNs (Ef/CBD-SD@DMNs) for deep transdermal delivery and enhanced melanoma therapy. As illustrated in Scheme 1, Ef/CBD-SD@DMNs are facilely fabricated by combining CBD solid dispersion (CBD-SD) and effervescent components (NaHCO_3_ + CaCO_3_) through the “one-step micro-molding” method. When pressed into the skin, Ef/CBD-SD@DMNs burst CO_2_ bubbles to push the CBD toward the tumors. With a sufficient supply of Ca^2+^ from CaCO_3_, CBD activates TRPV1 to induce melanoma apoptosis via the “Ca^2+^ influx-NFATc1-ATF3” pathway. Meanwhile, the effervescent components raise the intra-tumoral pH, leading to M1 repolarization of the TAMs and thereby relieving the immunosuppressive TME. Such a “one stone, two birds” effervescent design not only improves transdermal delivery of CBD but also creates favorable conditions for CBD to boost the anti-melanoma efficacy. This study focuses on the preparation, characterizations, transdermal delivery, tumoral penetration, antitumor efficacy, and TME characterizations by Ef/CBD-SD@MNs. This study has promising potential in using transdermal CBD delivery for melanoma therapy and provides a straightforward tool for transdermal therapy of skin tumors.

## Materials and methods

### Materials

CaCl_2_ (purity: 96%) and ethanol (purity: 98%) were purchased from Guangdong Xilong Scientific Co., Ltd. Cannabidiol (CBD; purity: 99%), polyvinylpyrrolidone (PVP; average Mw: 1,300,000, K88-96), NaHCO_3_ (purity > 99%), CaCO_3_ (purity: 98%; size: ≤ 50 nm), and coumarin 6 (C6) were purchased from Shanghai Aladdin Biochemical Technology Co., Ltd. Acetonitrile was purchased from Anhui Tedia High Purity Solvents Co., Ltd. Tween 80 (water content: ≤ 3%) was purchased from Guangzhou saiguo biotech Co., Ltd. Propidium Iodide was purchased from KeyGEN (Nanjing, China). Ultrapure water was acquired via ElixEssential system (Millipore, Shanghai, China). Other chemicals were used of analytical grade.

### Cell culture

Melanoma cells (B16-F10) were purchased from the National Collection of Authenticated Cell Cultures (Shanghai, China) and cultured in Dulbecco’s Modified Eagle’s Medium (DMEM) containing 10% fetal bovine serum (FBS), 1% penicillin (100 U/mL) and streptomycin (100 mg/mL) in a cell incubator (Thermo Fisher, USA) at 37℃ with 5% CO_2_ and 90% humidity. Calcium-free DMEM was purchased from Yuchun BioTech Co., Ltd. (Shanghai, China). 0.3 mmol/L, 2 mmol/L, and 10 mmol/L CaCl_2_ was added into calcium-free DMEM to obtain Ca^2+^ (low) DMEM, Ca^2+^ (medium) DMEM, Ca^2+^ (high) DMEM, respectively.

### Animals

Healthy male C57BL/6J mice weighing 20 ± 2 g were provided and raised by the Animal Experiment Center of Jiangsu Province Academy of Traditional Chinese Medicine, and all animal experiments met the ethical requirements stipulated by the National Experimental Animal Center.

### Preparation of Ef/CBD-SD@DMNs

20 mg of CBD, 111.11 mg of PVP, 20 mg of CaCO_3_, and 16.8 mg of NaHCO_3_ were dissolved in 1 mL of ethanol and strongly stirred at 600 rpm for 30 min. The mixture solution was cast into a microneedle mold (12 × 12 arrays; length 1000 μm; diameter 460 μm) and centrifuged at 4500 rpm for 15 min, followed by drying thoroughly at room temperature to obtain Ef/CBD-SD@DMNs. NaHCO_3_/CBD-SD@DMNs or CBD-SD@DMNs was prepared by the similar method mentioned above but without CaCO_3_ or CaCO_3_&NaHCO_3_.

### Characterizations of Ef/CBD-SD@DMNs

The DMNs were measured with an X-ray diffractometer (Rigaku, Japan) using Cu Kα radiation at a tube voltage of 40 kV, a current of 40 mA, and a scan 2θ angle range of 3 ~ 40°. The scanning speed was set at 0.1s/step with a step size of 0.02°. The DMNs samples to be tested were sprayed conductive coating and imaged using a scanning electron microscope (SEM, LEO 1530VP, Germany) equipped with an energy-dispersive X-ray spectroscopy (EDS, Germany). The DMNs were also cut into single rows and taken images using a fluorescence inverted microscope (IX73, Olympus, Japan). The mechanical property was tested by a pressure tester (Mark-10, USA), a cylindrical probe with a diameter of 10 mm compressed the microneedle arrays at a rate of 10.5 mm/min, and the force-displacement of the arrays was recorded to plotted the force-distance curve. The dynamic processes of Ef/CBD-SD@DMNs dissolving in different mediums were recorded by a fluorescence inverted microscope (IX73, Olympus, Japan).

### Isolation of mice skin

The mice were anesthetized with intramuscular administration of 50% ethyl carbamate solution (50 mg/kg), and the hair was shaved with an electric razor and then removed completely by a depilatory paste. After being sacrificed, the abdominal skin of the mice was isolated, followed by removing the subcutaneous tissues and washing it with saline thrice.

### Skin permeation

Pig skin was purchased from a nearby wet market. The DMNs were pressed into the excised pig skin for 5 min. After removal of the DMNs, a part of the treated skin was fixed with 4% paraformaldehyde for 48 h, dehydrated, embedded in paraffin, and prepared the section, successively. After being stained with hematoxylin and eosin (H&E), the slice was observed with a microscope (IX73, Olympus, Japan).

### Chromatographic conditions of CBD

CBD was determined at 37 °C by an Agilent 1260 Infinity instrument (Agilent Technologies Inc., Santa Clara, CA, USA) with the ZORBAX SB-C18 column (250 × 4.6 mm, 5 μm) eluted with deionized water-acetonitrile (35/65, v/v) at a flow of 1 mL/min and a detection wavelength at 220 nm. The linear regression equation with drug concentration as the independent variable and peak area as the dependent variable was y = 28.407x + 65.452 (r = 0.9998) in the range of 2 ~ 512 µg/mL.

### Dissolution test

100 mL of phosphate buffer containing Tween 80 (1%) was degassed and added to a dissolution tester (ZRS-8G, Tianjin, China). The DMNs were separately added into the medium with different pH values, followed by stirring the medium at 100 rpm at (37.0 ± 0.5) ℃. The mediums were collected at the predetermined time intervals, the same volume of fresh medium was immediately replenished. The samples were centrifuged at 13,000 rpm for 15 min and the supernatant was harvested to detect by HPLC. Cumulative dissolution amount (µg) = C_d_ × V_m_, where C_d_ and V_m_ are the accumulative content of CBD in the dissolution cups and the volume of the medium, respectively. Every experiment was performed in triplicate.

### Transdermal Penetration and Retention in vitro

A piece of freshly-isolated mice abdominal skin was fixed in a Franz diffusion instrument (TK-24BL, Shanghai, China) with the cross-sectional area of the Franz diffusion cell of 0.785 cm^2^. The stratum corneum of the skin was facing the supply cell and the PBS solutions with different pH values were in the receiving cell. The DMNs were rapidly pressed into the skin, followed by stirring the receiving medium at 250 rpm at 37 °C. The mediums were collected at the predetermined time intervals. At the end of the transdermal studies, the skin was rinsed with saline thrice and grinded into homogenates solution (with 1% Tween 80 PBS), followed by centrifuging at 13,000 rpm for 15 min. The supernatant was harvested to detect by HPLC. Transdermal flux was calculated as the following equation. Transdermal flux (µg/cm^2^) = C_t_/S_F_, where C_t_ and S_F_ are the accumulative content of CBD in the receiving cell and the cross-sectional area of the Franz diffusion cell, respectively. Retention (µg/cm^2^) = C_r_/S_F_, where C_r_ and S_F_ are the content of CBD in the skin and the cross-sectional area of the Franz diffusion cell, respectively.

### Cytotoxicity

Ten hundred thousand B16-F10 cells were seeded in the 96-well plates and cultured overnight. The groups set as follows, CBD (Ca^2+^ (low)), CBD (Ca^2+^ (medium)), and CBD (Ca^2+^ (high)) at a CBD concentration ranging from 0 ~ 8 µg/mL. After being co-incubated for 24 h, the cells were stained with 10 µL of MTT (5 mg/mL) PBS solution for 4 h and dissolved with 150 µL DMSO. The absorbance in each well was determined at 450 nm with a microplate reader (MULTISKAN GO, Thermo Fisher, USA). The value of IC_50_ was calculated by GraphPad Prism 8. Cell activity was calculated according to the following formula.

$$\text{C}\text{e}\text{l}\text{l} \text{a}\text{c}\text{t}\text{i}\text{v}\text{i}\text{t}\text{y} \left(\text{\%}\right) = \frac{{\text{O}\text{D}}_{\text{A}\text{d}\text{m}\text{i}\text{n}\text{i}\text{s}\text{t}\text{r}\text{a}\text{t}\text{i}\text{o}\text{n}} - {\text{O}\text{D}}_{\text{B}\text{l}\text{a}\text{n}\text{k}}}{{\text{O}\text{D}}_{\text{C}\text{o}\text{n}\text{t}\text{r}\text{o}\text{l}} - {\text{O}\text{D}}_{\text{B}\text{l}\text{a}\text{n}\text{k}}} \times 100\text{\%}$$

Where OD_Administration_, OD_Control_, and OD_Blank_ represent the absorbance of the administration group, control group, and blank group, respectively.

### Intracellular calcium determination

Five hundred thousand B16-F10 cells were seeded in the 6-well plates and cultured overnight. CBD (Ca^2+^ (low)), CBD (Ca^2+^ (medium)), and CBD (Ca^2+^ (high)) at a CBD concentration of 2.5 µg/mL were co-incubated with the cells for 2 h. And then, the cells were stained with Fluo-4 AM (1.5 µmol/L) in dark for 30 min, followed by washing with cold PBS and digesting with EDTA-free trypsin. The intracellular fluorescence was detected by a flow cytometer (Beckman Coulter, USA). Each experiment was done in triplicate.

### Apoptosis analysis

Five hundred thousand B16-F10 cells were seeded in the 6-well plates and cultured overnight. CBD (Ca^2+^ (low)), CBD (Ca^2+^ (medium)), and CBD (Ca^2+^ (high)) at a CBD concentration of 2.5 µg/mL were co-incubated with the cells for 2 h. After being digested with EDTA-free trypsin, the cell suspension was stained with an appropriate volume of Annexin V-FITC and Propidium Iodide (KeyGEN, China) in dark for 10 min, followed by assaying using a flow cytometer (Beckman Coulter, USA) immediately. Each experiment was done in triplicate.

### Immunofluorescence staining in vitro

Five hundred thousand B16-F10 cells were seeded in the circle microscope cover glasses and cultured overnight. CBD (Ca^2+^ (high)) at a CBD concentration of 0, 2.5, and 4 µg/mL were co-incubated with the cells for 2 h. After the removal of the medium, the cover glasses were fixed with 4% paraformaldehyde. Then, the cells were then permeabilized with Triton X-100 (0.1%, v%) for 15 min, followed by the addition of a blocking solution consisting of bovine serum albumin (BSA, 1%) and incubated at room temperature for 30 min. Next, all samples were incubated with the primary antibodies at room temperature for 12 h, followed by staining with the corresponding secondary antibodies in dark for 1 h. The primary antibodies included ATF3(ab207434, Abcam) and NFATc1(sc-7294, SANTA CRUZ). The following secondary antibodies included IgG (H + L) Fluor 555-conjugated (A0460, Beyotime) and IgG (H + L) Fluor 647-conjugated (A0468, Beyotime). Nuclei were labeled with DAPI (BL105A, Biosharp) at room temperature for 5 min. After washing with PBS thoroughly, immunofluorescence images were then acquired with a multiphoton confocal microscopy (STELLARIS 8 DIVE, Leica).

### Construction of B16-F10 xenograft-bearing mice

Two hundred microliters of B16-F10 cell suspension at a density of 1 × 10^4^ cells/µL was injected into the right back of C57BL/6J mice. The body weight and survival of mice were recorded daily. Tumor volume (mm^3^) was calculated according to the following formula,

$$\text{T}\text{u}\text{m}\text{o}\text{r} \text{v}\text{o}\text{l}\text{u}\text{m}\text{e} = \frac{\text{l}\text{e}\text{n}\text{g}\text{t}\text{h} \times {\text{w}\text{i}\text{d}\text{t}\text{h}}^{2}}{2}.$$

Where length and width are the long diameter and short diameter of the tumor. Once the tumor size exceeded 50 mm^3^, transdermal therapy was performed.

### Antitumor Efficacy in vivo

The B16-F10 xenograft tumor-bearing mice were randomly divided into 5 groups (n = 5) as follows, control, CBD (i.p.), CBD-SD@DMNs, NaHCO_3_/CBD-SD@DMNs, and Ef/CBD-SD@DMNs. All the DMNs were administrated at a CBD dose of 5 mg/kg once every day. During the treatments, the body weight, tumor volume, and survival of the mice were recorded every day. After 7 days of the treatments, the mice were killed humanely, followed by harvesting the blood, tumors, hearts, livers, spleens, lungs, and kidneys. The tumor index (mg/g) was calculated according to the following formula. Tumor index = W_t_/W_b_, where W_t_ and W_b_ are the tumor weight and the body weight, respectively. To reflect the change in tumor volume before and after treatment, V_15_/V_9_ (mm^3^/mm^3^) was calculated, namely the ratio of tumor volume on day 15 to that on day 9. The serum was prepared to quantify inducible nitric oxide synthase (iNOS), interleukin-12P40 (IL-12P40), interleukin-6 (IL-6), interferon-gamma (IFN-*γ*), tumor necrosis factor-α (TNF-α), interleukin-10 (IL-10), arginase 1 (Arg-1), and transforming growth factor-β1 (TGF-β1) using the corresponding ELISA kits under the manufacturer’s protocol. Besides, the paraffin-embedded tumor sections were stained with H&E according to classic protocol.

### Immune cells determination in tumors

The harvested tumors of the mice were cut into pieces in DMEM medium containing 2% FBS on ice and then treated with 5 volume equivalent trypsin at 37℃ for 60 min. The samples were filtered and centrifuged at 600 g for 5 min. The cells were washed with PBS containing 2% FBS and co-incubated with sterile ZombieNIR dye and Fc receptor blocker at 4℃ for 15 min. Next, the cells were stained with the primary antibodies in dark for 30 min, including anti-mouse CD86-PE antibody (B334834, Biolegend), anti-mouse CD206-APC (B354282, Biolegend), anti-mouse F4/80-PE/Cyanine-7 (B342137, Biolegend), anti-mouse CD45-Brilliant Violet 421 (B343559, Biolegend), anti-mouse CD3-PE (B341466, Biolegend), anti-mouse CD4-FITC (B269033, Biolegend), anti-mouse CD8a-APC (B348048, Biolegend), anti-mouse CD11c-APC (B339313, Biolegend), anti-mouse I-A/I-E (MHC II)-PE/Cyanine-7 (B337001, Biolegend) and anti-mouse CD11b-FITC (B349919, Biolegend). After rinsing excess antibodies, the cells were immediately assessed by a flow cytometer (Beckman Coulter, USA).

### Immunofluorescence staining in vivo

A part of the tumors from each group was successively deparaffinized, permeabilized, and blocked with 1% BSA as aforementioned. Next, all the samples were incubated with primary antibodies at 37 °C for 1 h. The corresponding secondary antibodies were stained in dark for 1 h. The antibodies included TUNEL (ab66110, Abcam), ATF3(ab207434, Abcam), NFATc1(sc-7294, SANTA CRUZ), and Foxp3-PE (B325779, Biolegend). Nuclei were labeled with DAPI for 5 min. After washing with PBS thoroughly, the fluorescent images were acquired with a CLSM (FV101i, OLYMPUS) and quantified by Image J software.

### Transdermal and tumoral penetration

The B16-F10 xenograft tumor-bearing mice were randomly divided into 5 groups as follows, C6 (i.p.), C6 solution, C6-SD@DMNs, NaHCO_3_/C6-SD@DMNs, and Ef/C6-SD@DMNs. Each group of mice was administrated at a C6 dosage of 5 mg/kg and killed humanely after 1 h. Each tumor was collected and prepared into frozen sections. The nuclei were labeled with DAPI for 5 min. After the removal of excess dyes, the tumor sections were fixed with 4% paraformaldehyde, sealed with an antifade mounting medium, and immediately observed by a CLSM (FV101i, OLYMPUS).

### Skin irritation

The right-back skin of male C57BL/6J mice was depilated and disinfected. Then the microneedles were pressed to the skin for 5 min once every day for 7 days. The surface of the treated skin was observed daily.

### Data analysis

All Data presented in this study were shown as a mean ± standard deviation (SD). Statistical tests were performed by GraphPad prism 8.0 statistical software. ANOVA was employed to evaluate the statistical significance. Statistical significance was defined as the values of *P < 0.05, **P < 0.01, and ***P < 0.001.

## Results

### Fabrication and characterizations of Ef/CBD-SD@DMNs

**Fig. 1:**
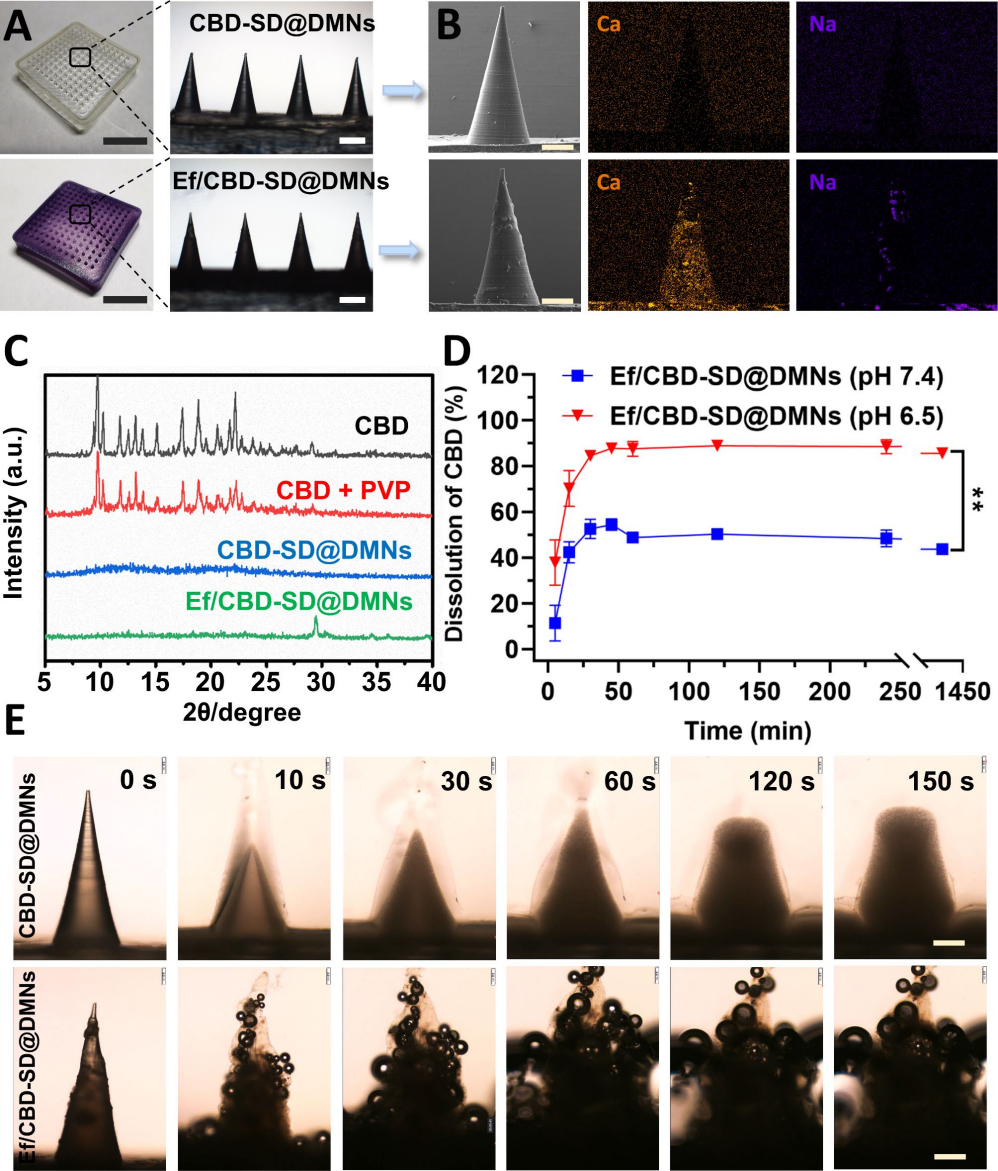
Preparation and characterizations of Ef/CBD-SD@DMNs. (A) Pictures of the patches (bar, 1 cm). The right part is the corresponding optical micrograph (bar, 500 μm). (B) SEM images and EDS analysis of CBD-SD@DMNs (upper) and Ef/CBD-SD@DMNs (lower). The bar is 250 μm. (C) XRD spectrum of CBD-associated formulations. (D) Cumulative dissolution of CBD from Ef/CBD-SD@DMNs at different pH values. Data are represented as mean ± SD, n = 6. **P < 0.01. (E) CO_2_ bubbles production and dissolution of the arrays in different pH environments within 150 s. The bar is 200 μm

Ef/CBD-SD@DMNs were facilely fabricated by the “one-step micro-molding” method, as presented in Scheme 1A. As shown in Fig. 1A, the CBD-SD@DMNs transparent patch had 12 × 12 uniform conical arrays with a base diameter of ~ 460 μm and a height of ~ 1000 μm. The morphology and corresponding energy dispersive X-ray spectroscopy (EDS) mapping analysis of Ef/CBD-SD@DMNs exhibited a uniform dispersion of NaHCO_3_ and CaCO_3_ in the array (Fig. 1B), which resulted in a slightly-rough surface in comparison with that of CBD-SD@DMNs. According to the X-ray diffraction (XRD) of Ef/CBD-SD@DMNs, the characteristic diffractive peaks ascribed to both CBD and PVP disappeared (Fig. 1C). Due to the SD and effervescent design, cumulative dissolution of CBD from Ef/CBD-SD@DMNs reached ~ 90% within 30 min in a mildly-acidic environment, which was 1.73 times that in the physiological environment (Fig. 1D). In contrast, due to the lack of the effervescent components, the 24 h-cumulative dissolution rate of CBD-SD@DMNs was about 50%, which is not dependent on the pH environment (Figure S1). As shown in Fig. 1E, Ef/CBD-SD@DMNs produced plenty of bubbles in PBS (pH 6.5) within 60 s(Fig. 1E).

### Penetration and transdermal delivery in vitro

**Fig. 2:**
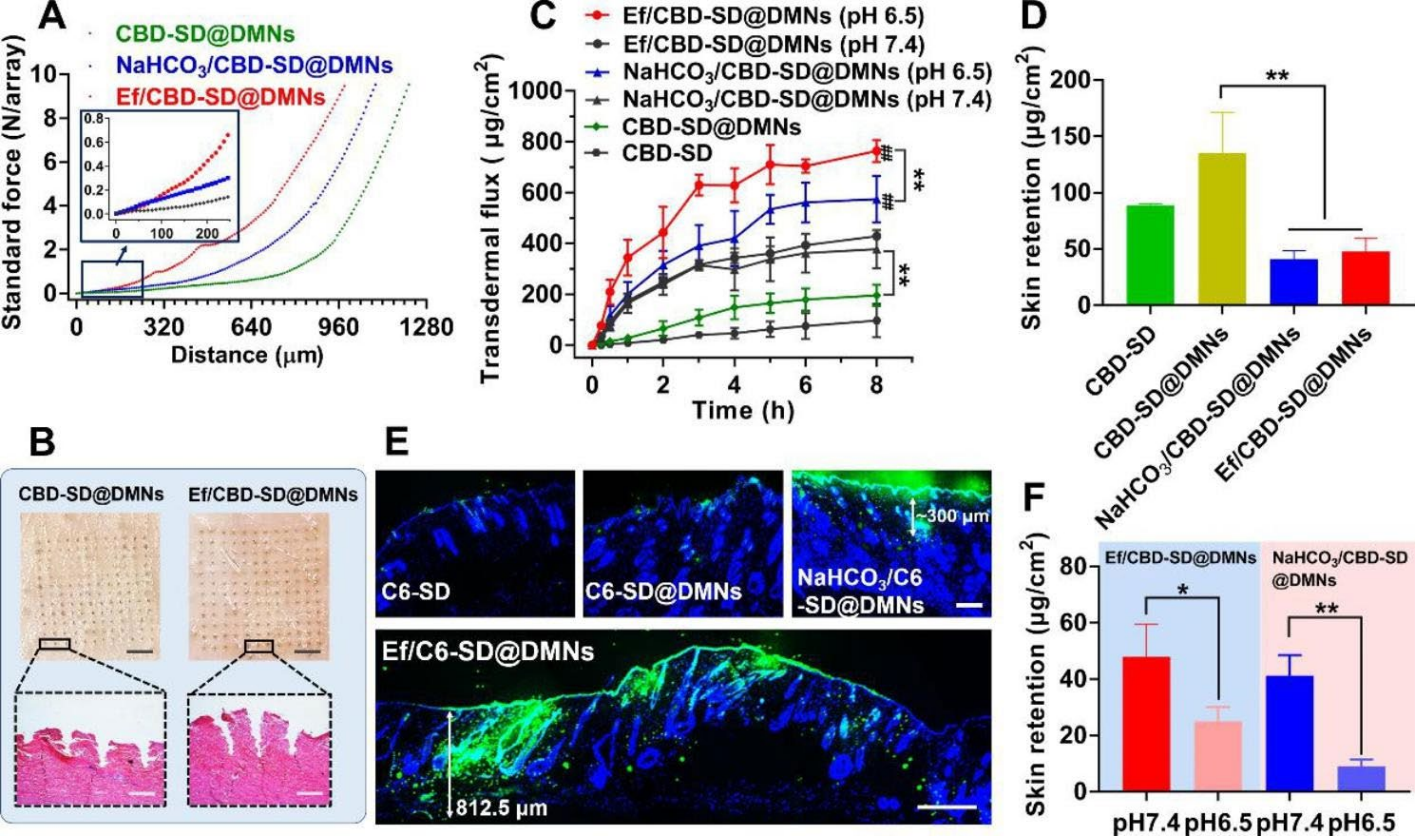
In vitro penetration and drug diffusion. (A) Force-distance curve of different CBD-SD@DMNs. (B) Images of porcine skins treated with different formulations and the H&E-stained sections. The bars are 2.5 mm (upper) and 200 μm (lower), respectively. (C) Dynamic transdermal flux of various formulations within 8 h. Data are represented as mean ± SD, n = 6. **P < 0.01; ^##^P < 0.01 vs. the corresponding formulation at pH 7.4. (D) Skin retention of CBD after 8 h of treatments with different formulations. Data are represented as mean ± SD, n = 6. **P < 0.01. (E) Fluorescence images of various C6-labeled formulations-treated skins. The blue is DAPI and the green is the C6 signal. The bar is 200 μm (upper) and 450 μm (lower). (F) Skin retention of CBD after 8 h of treatments with the two effervescent DMNs at different pH values. Data are represented as mean ± SD, n = 6. *P < 0.05; **P < 0.01

As shown in Fig. 2A, the insertion force of the three SD@DMNs reached at least 10 N/array, and Ef/CBD-SD@DMNs exhibited the strongest hardness. As illustrated in the HE-stained images (Fig. 2B), Ef/CBD-SD@DMNs successfully pierced the pig skin and reached the epidermis (> 700 μm), which is deeper than CBD-SD@DMNs. In addition, Ef/CBD-SD@DMNs were able to pierce the three-layer parafilm, with a significantly higher penetration rate than CBD-SD@DMNs (Figure S2). To investigate the transdermal performance of Ef/CBD-SD@DMNs, fluorescence distribution and drug quantification in the porcine skin were studied, respectively. As demonstrated in Fig. 2C, the 8 h-cumulative transdermal flux of CBD-SD@DMNs was ~ 120 µg/cm^2^, significantly higher than that of CBD-SD. Similarly, the dynamic transdermal flux of NaHCO_3_/CBD-SD@DMNs at pH 7.4 was significantly improved compared to that of CBD-SD@DMNs. Notably, the transdermal flux of NaHCO_3_/CBD-SD@DMNs and Ef/CBD-SD@DMNs at pH 6.5 was noticeably improved in comparison to their behavior at pH 7.4. The 8 h-cumulative transdermal flux of Ef/CBD-SD@DMNs reached ~ 710 µg/cm^2^, remarkably higher than that of NaHCO_3_/CBD-SD@DMNs. However, no statistical difference in transdermal behavior was observed between NaHCO_3_/CBD-SD@DMNs and Ef/CBD-SD@DMNs at pH 7.4. Undesired retention in the skin is not favorable to the transdermal delivery, nor is it for tumoral penetration. As shown in Fig. 2D, the 8 h-cumulative skin retention of CBD-SD@DMNs at pH 7.4 was ~ 130 µg/cm^2^, which is significantly higher than that of both NaHCO_3_/CBD-SD@DMNs and Ef/CBD-SD@DMNs. The skin retention of the two effervescent DMNs considerably decreased at pH 6.5 than that at pH 7.4 (Fig. 2F). In addition, after pressing Ef/C6-SD@DMNs into the skin, the C6 signal was observed at a distance of ~ 812.5 μm from the stratum corneum, which is notably deeper than the NaHCO_3_/C6-SD@DMNs (only ~ 300 μm), as well as the C6-SD@DMNs. These findings further indicate that the effervescent components generating CO_2_ bubbles actively push the drug towards efficient transdermal delivery. Moreover, considering possible skin irritation, the safety of Ef/CBD-SD@DMNs was evaluated. As shown in Figure S3, Ef/CBD-SD@DMNs inserted into the skin once every day for 7 days did not cause any wounds.

### Efficacy and mechanism of anti-melanoma in vitro

**Fig. 3:**
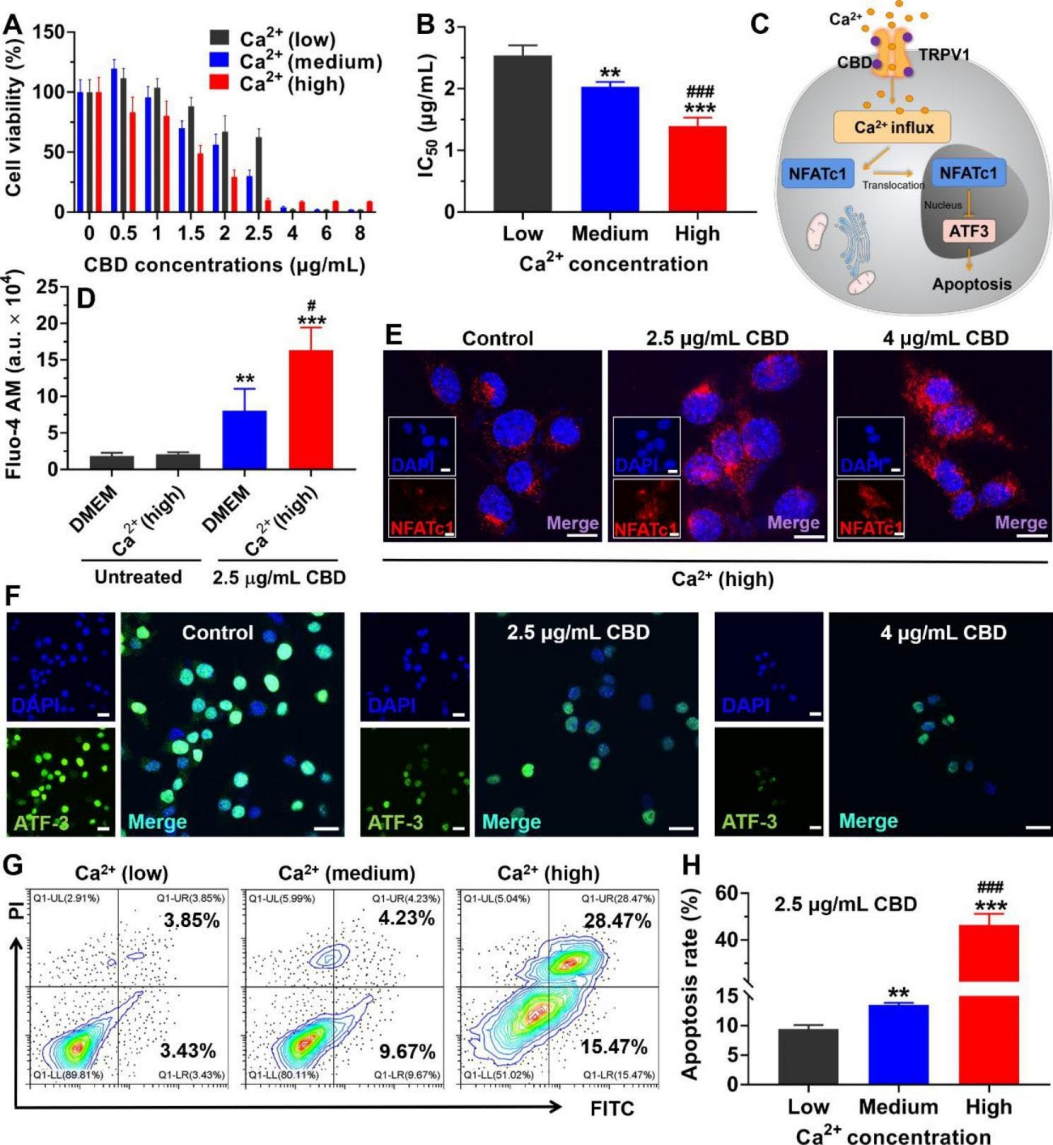
In vitro anti-melanoma mechanism. (A) B16-F10 cell viability and (B) the IC_50_ after incubation with CBD-SD. Different concentrations of Ca^2+^ were added in the culture medium. Data are represented as mean ± SD, n = 4. **P < 0.01. ***P < 0.001 vs. Low; ^###^P < 0.001 vs. Medium. (C) Schematic illustration of the “Ca^2+^ influx-NFATc1-ATF3” pathway. (D) Fluorescence intensity of Fluo-4 AM in B16-F10 after different treatments. Data are represented as mean ± SD, n = 4. **P < 0.01. ***P < 0.001 vs. corresponding untreated group; ^#^P < 0.05 vs. DMEM (2.5 µg/mL CBD). (E) Immunofluorescence staining of NFATc1 (red) of B16-F10 after different treatments. The bar is 10 μm. (F) Immunofluorescence staining of ATF3 (green) of B16-F10 after different treatments. The bar is 20 μm. (G) Representative flow cytometric and (H) quantitative analysis of apoptosis of B16-F10 cells after different treatments. Data are represented as mean ± SD, n = 4. **P < 0.01. ***P < 0.001 vs. Low; ^###^P < 0.001 vs. Medium

To figure out whether the incorporation of CaCO_3_ into Ef/CBD-SD@DMNs could enhance the anti-melanoma effect, the inhibition of CBD-SD against B16-F10 cells cultured with different calcium concentrations was investigated. As shown in Fig. 3A and B, treatment with CBD-SD significantly suppressed the viability of B16-F10 cells cultured with high calcium concentrations once the CBD concentration exceeded 1.5 µg/mL, with an IC_50_ of ~ 2.03 µg/mL, which is significantly lower than that of the medium- and low-calcium groups. As reported previously [3], increased calcium influx triggered the nuclear translocation of NFATc1, subsequently downregulated the transcription factor ATF3, and consequently led to melanoma apoptosis (Fig. 3C). To elucidate the anti-melanoma mechanism of the CBD-SD and calcium ions, the “TRPV1-NFATc1-ATF3” pathway was studied under different calcium concentrations. As shown in Fig. 3D, the intracellular Fluo-4 AM fluorescence had no significant change regardless of whether B16-F10 cells were cultured with the normal DMEM medium or supplemented with exogenous calcium ions. In contrast, after the treatment with CBD-SD at a CBD concentration of 2.5 µg/mL, Ca^2+^ influx noticeably enhanced with the exogenous calcium increased (Fig. 3D). The NFATc1 was distributed in the cytoplasm of the untreated B16-F10 cells. Once treated with CBD-SD, the nuclear translocation of NFATc1 presenting as the overlap of the red and blue signal was easily observed (Fig. 3E). Under the high-calcium conditions, the expression of ATF3 in the nucleus was significantly reduced with the treatment of CBD-SD (Fig. 3F). As demonstrated in Fig. 3G H, high-calcium culture conditions significantly improved the apoptosis-inducing effect of CBD-SD on B16-F10 cells.

### Anti-melanoma efficacy in vivo

**Fig. 4:**
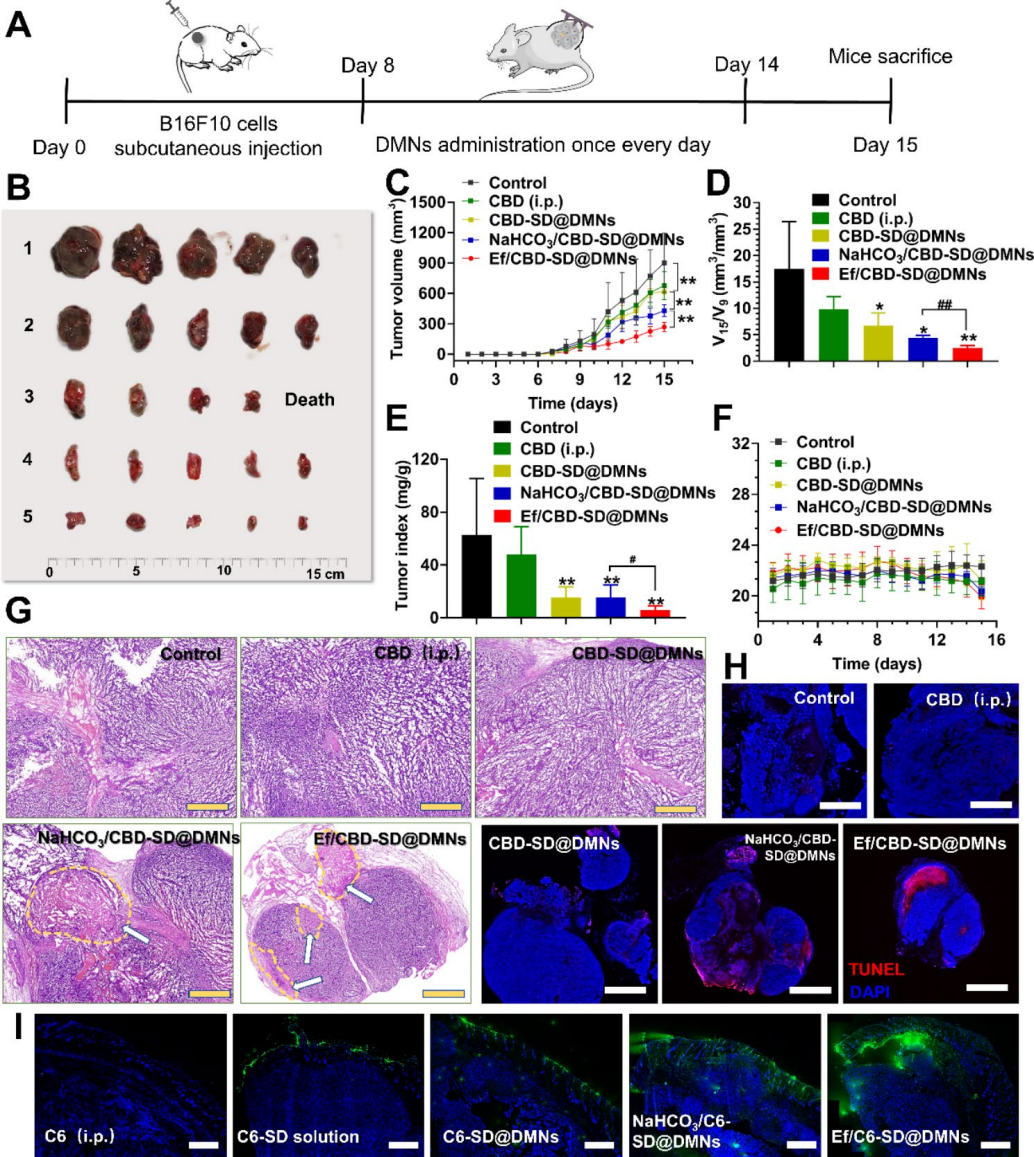
Anti-melanoma efficacy in vivo. (A) Schematic illustration of establishment of tumor-bearing mice model and therapeutic regimens. (B) Ex vivo tumors at the end of the treatments. (1. Control, 2. CBD (i.p.), 3. CBD-SD@DMNs, 4. NaHCO_3_/CBD-SD@DMNs, and 5. Ef/CBD-SD@DMNs.) (C) Tumor growth curve of mice treated with various treatments. Data are represented as mean ± SD, n = 5. *P < 0.05. **P < 0.01. ***P < 0.001. (D) The ratio of tumor volume on day 15 to day 9. Data are represented as mean ± SD, n = 5. *P < 0.05. **P < 0.01. ***P < 0.001. (E) Tumor index and (F) body weight of tumor-bearing mice treated with different treatments. Data are represented as mean ± SD, n = 5. *P < 0.05. **P < 0.01. ***P < 0.001. (G) H&E-stained tumor sections after different treatments. The bar is 500 μm. (H) Immunofluorescence staining of TUNEL of tumor sections after different treatments. The bar is 1 mm. (I) Fluorescence images of tumor sections of mice treated with various C6-labeled formulations. The bar is 2 mm

After the establishment of the B16-F10 melanoma-bearing mice model, various DMNs were administrated at a CBD dosage of 5 mg/kg once every day for 7 days (Fig. 4A). As a control, the mice were intraperitoneally injected with CBD (CBD (i.p.), 5 mg/kg) once every two days [41]. As shown in Fig. 4B, the treatment of CBD (i.p.) slightly inhibited the growth of melanoma, while the volume of the tumors of mice treated with the three DMNs was significantly shrunk compared to the control group. According to the tumor growth curve (Fig. 4C), the average volume of tumors of mice treated with NaHCO_3_/CBD-SD@DMNs was remarkably smaller than that of CBD-SD@DMNs. Ef/CBD-SD@DMNs exhibited the most significant inhibition against melanoma among all the groups. The V_15_/V_9_, representing the growth rate of melanoma, was noticeably retarded after the treatment with the three DMNs. As expected, Ef/CBD-SD@DMNs presented a significantly lower V_15_/V_9_ than NaHCO_3_/CBD-SD@DMNs (Fig. 4D). Besides, the treatment with Ef/CBD-SD@DMNs resulted in the lowest tumor index among all the groups (Fig. 4E). The body weight of the mice treated with different formulations showed no significant fluctuation (Fig. 4F), indicating acceptable systemic safety. According to the HE-stained and TUNEL-stained tumor sections, the two effervescent CBD-SD@DMNs groups not only resulted in obvious necrosis but also induced substantial tumor cell apoptosis (Fig. 4G H). According to our hypothesis, effervescent DMNs improve anti-melanoma efficacy by CO_2_ bubbles-thrusted transdermal and tumoral penetration. As shown in Fig. 4I, the penetration of the two effervescent DMNs was notably deeper than that of CBD-SD@DMNs. After treatment with Ef/C6-SD@DMNs, intra-tumoral C6 distribution was obviously higher than NaHCO_3_/C6-SD@DMNs.

### Engineering of the TME

**Fig. 5:**
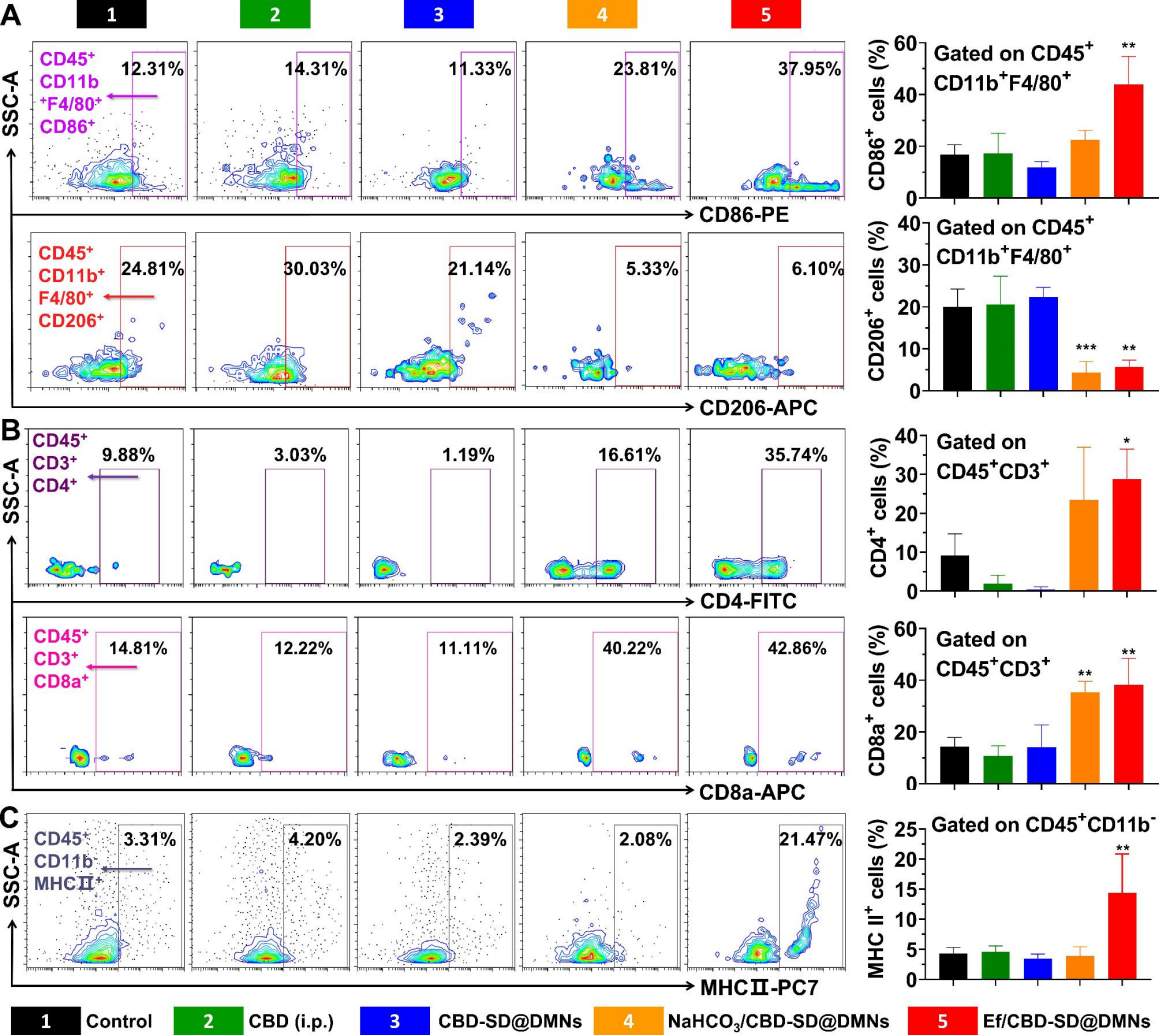
Influence on the tumoral immune cells. (A) Representative flow cytometric analysis and quantitative analysis of M1-like macrophages (CD86^+^) and M2-like macrophages (CD206^+^) gated on CD45^+^CD11b^+^F4/80^+^ cells after different treatments. Data are represented as mean ± SD, n = 5. **P < 0.01. ***P < 0.001. (B) Representative flow cytometric analysis and quantitative analysis of helper T cells (CD4^+^) and cytotoxic T cells (CD8^+^) gated on CD45^+^CD3^+^ cells after different treatments. Data are represented as mean ± SD, n = 5. *P < 0.05. **P < 0.01. (C) Representative flow cytometric analysis and quantitative analysis of dendritic cell (MHC II^+^) gated on CD45^+^CD11b^−^ cells after different treatments. Data are represented as mean ± SD, n = 5. **P < 0.01

As shown in Fig. 5A, neither CBD (i.p.) nor CBD-SD@DMNs treatment influenced the population of CD45^+^CD11b^+^F4/80^+^CD86^+^ or CD45^+^CD11b^+^F4/80^+^CD206^+^ cells, which are ascribed to the M1 and M2 TAMs, respectively [42]. Notably, Ef/CBD-SD@DMNs could not only increase M1 TAMs but also decrease M2 TAMs in the TME. Likewise, NaHCO_3_/CBD-SD@DMNs could also decrease the percentage of M2 TAMs. Such similar H^+^ elimination-resulted TAMs repolarization is in accordance with a previous report [38]. Likewise, the treatment with Ef/CBD-SD@DMNs led to a higher population of CD45^+^CD3^+^CD4^+^, as well as CD45^+^CD3^+^CD8^+^ cells; however, the CBD-SD@DMNs did not change the percentage of CD45^+^CD3^+^CD4^+^ or CD45^+^CD3^+^CD8^+^ cells (Fig. 5B), indicating that effervescent component is a potential reason for enhanced infiltration of T cells. Besides, Ef/CBD-SD@DMNs was even able to reduce the abundance of regulatory T cells (Foxp3^+^ area) in the TME (Figure S4). The percentage of T cells in the spleen was not changed noticeably after treatments with different DMNs (Figure S5). Although the recruitment of DCs precursors from peripheral circulation into certain tissues is dependent on Ca^2+^ signaling [43], whether sufficient Ca^2+^ influx can increase the abundance of intra-tumoral mature DCs remains unclear. As displayed in Fig. 5C, Ef/CBD-SD@DMNs significantly increased the abundance of CD45^+^CD11b^−^MHC II^+^ cells but NaHCO_3_/CBD-SD@DMNs failed, suggesting that the introduction of exogenous calcium to the tumors also increases DCs in the TME.

**Fig. 6:**
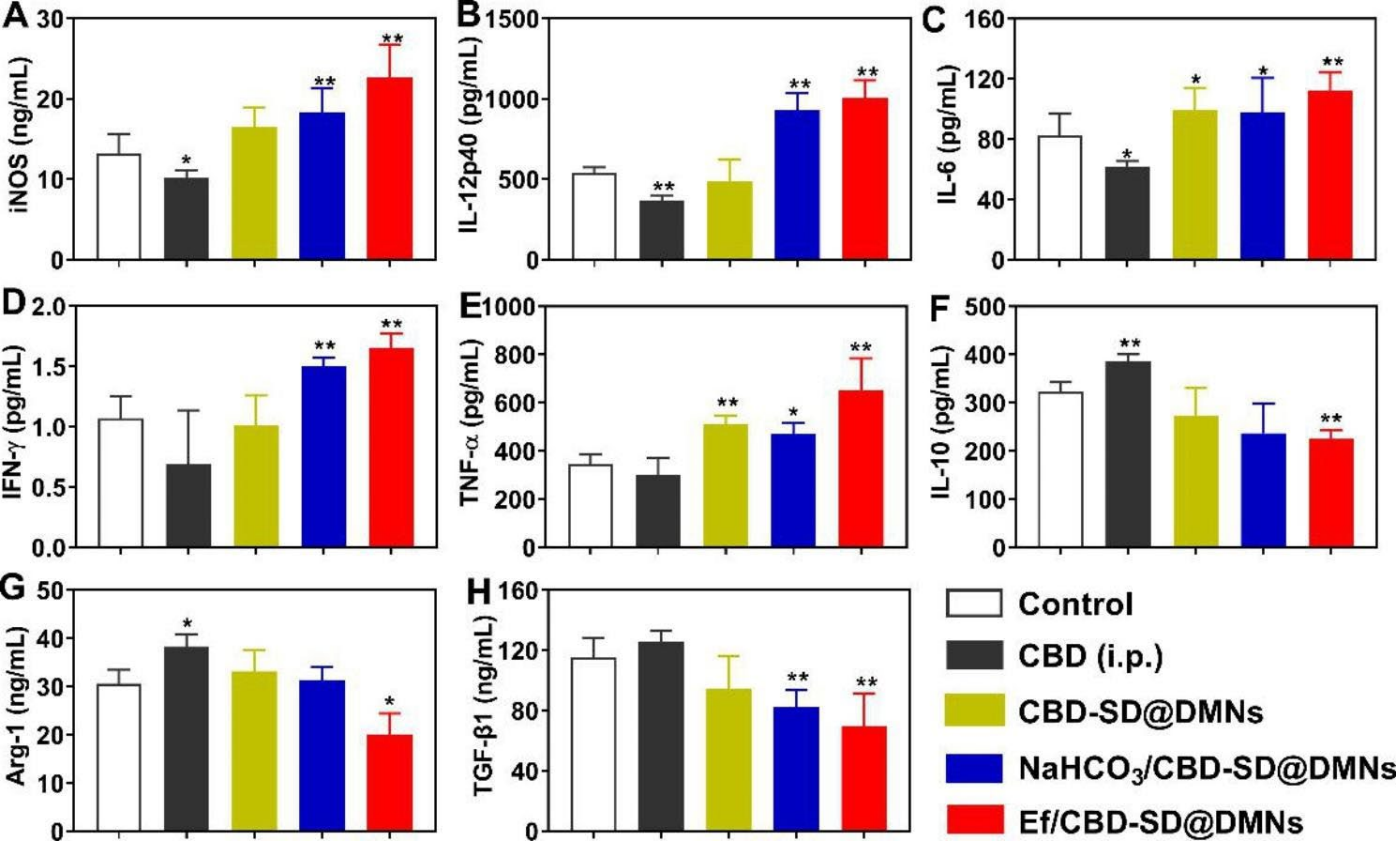
Serum cytokine detection. Changes in serum (A) iNOS, (B) IL-12p40, (C) IL-6, (D) IFN-γ, (E) TNF-α, (F) IL-10, (G) Arg-1, and (H) TGF-β1 after different treatments. Data are represented as mean ± SD, n = 5. *P < 0.05. **P < 0.01 vs. Control

As shown in Fig. 6A ~ 6 C, the serum iNOS, IL-12p40, and IL-6 (considered Th1 cytokines) of mice treated with CBD (i.p.) were decreased significantly compared with the control group, which might be associated with the anti-inflammatory effects of CBD. In contrast, NaHCO_3_/CBD-SD@DMNs and Ef/CBD-SD@DMNs upregulated the level of iNOS, IL-12p40, IL-6, IFN-γ, and TNF-α, which could trigger pro-inflammatory effects and consequently activate the TME (Fig. 6A ~ 6E). Moreover, Ef/CBD-SD@DMNs also downregulated serum levels of IL-10, Arg-1, and TGF-β1 (Fig. 6F ~ 6 H), which can alleviate immunosuppression.

### Ca^2+^ influx-NFATc1-ATF3 signaling in vivo

**Fig. 7:**
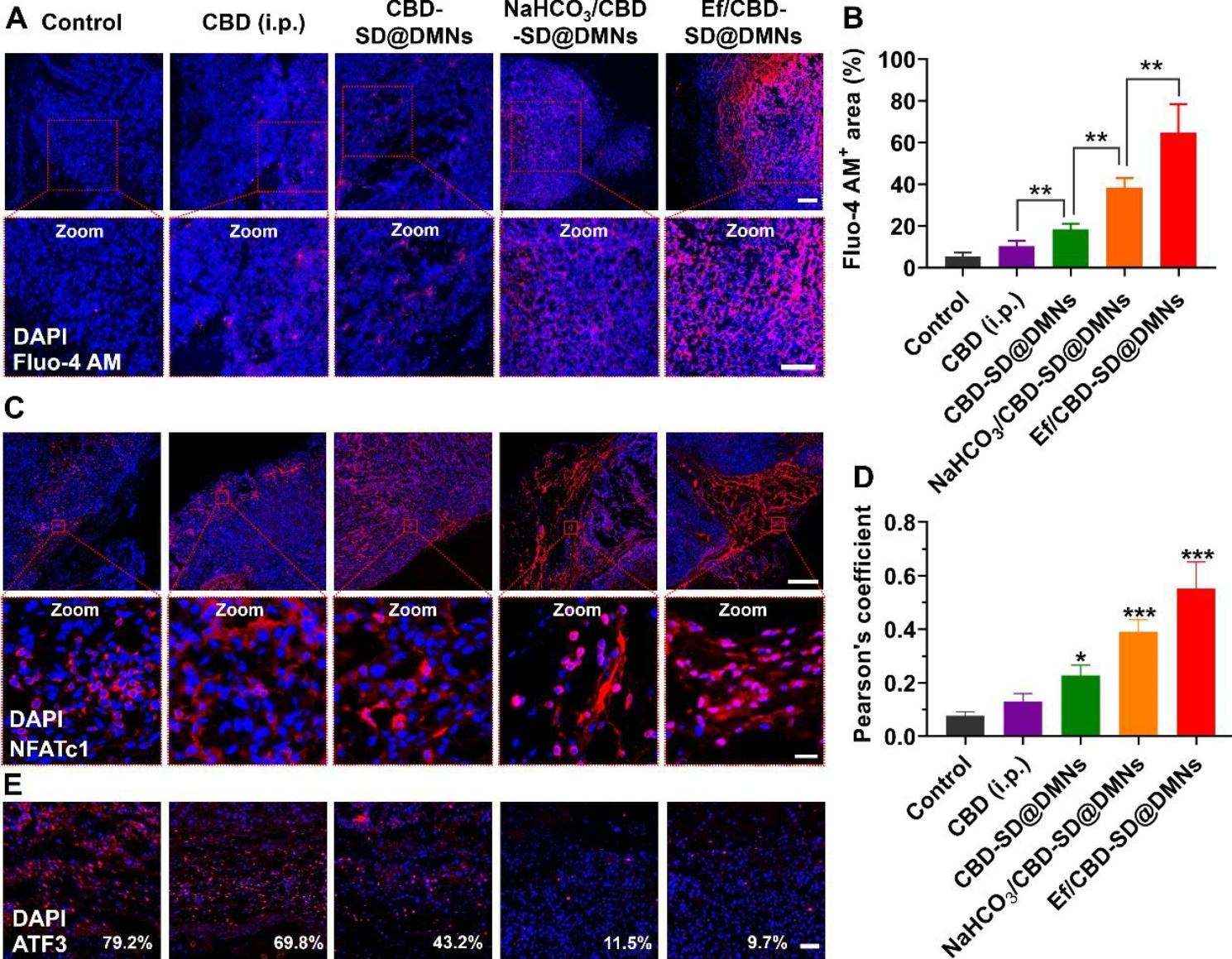
Validation of Ca^2+^ influx-NFATc1-ATF3 signaling inside the tumors. (A) Immunofluorescence staining and (B) quantitative analysis of Fluo-4 AM in the tumors after different treatments. The bar is 100 μm. Data are represented as mean ± SD, n = 5. **P < 0.01. (C) Immunofluorescence staining and (D) quantitative analysis of NFATc1 in the tumors after different treatments. The bar is 400 μm (upper) and 40 μm (lower). Data are represented as mean ± SD, n = 5. *P < 0.05; ***P < 0.001 vs. Control. (E) Immunofluorescence staining of ATF3 in the tumors after different treatments. The bar is 50 μm

As shown in Fig. 7A and B, CBD-SD@DMNs significantly increased Ca^2+^ influx in the tumor compared to the CBD (i.p.) group. The NaHCO_3_/CBD-SD@DMNs group exhibited remarkably higher Fluo-4 AM fluorescence than the CBD-SD@DMNs group, and mice treated with Ef/CBD-SD@DMNs had red fluorescence distributed almost throughout the tumor section, possibly due to calcium carbonate in the effervescent components. Immunofluorescent staining revealed nuclear translocation of NFATc1 triggered by Ca^2+^ influx (Fig. 7C and D). The NFATc1 was almost completely transferred into the nucleus in the Ef/CBD-SD@DMNs group, where the Pearson’s coefficient of the two fluorescence reached ~ 0.6, significantly higher than the NaHCO_3_/CBD-SD@DMNs and CBD-SD@DMNs group. According to the trend, the nucleus translocation of the NFATc1 is related to the amount of intracellular CBD. Afterward, the expression of ATF3 inside the tumors of mice treated with different formulations was investigated. As shown in Fig. 7E, the ATF3-positive area of tumor sections in the two effervescent CBD-SD@DMNs was significantly lower than that in the CBD-SD@DMNs group.

## Discussion

In this study, we successfully fabricated Ef/CBD-SD@DMNs using a one-step micro-molding method. To ensure a good dispersibility and hardness, PVP was employed as the matrix of both SD and DMNs, while the effervescent design significantly improved the dissolution of Ef/CBD-SD@DMNs in mildly-acidic environments through producing CO_2_ bubbles, which may increase the penetration of CBD into tumors. The addition of effervescent ingredients resulted in a slightly-rough surface and a purple color change, probably due to the color reaction of CBD in the weak base environment. XRD analysis indicated that the CBD in Ef/CBD-SD@DMNs was probably in an amorphous form, which has better dissolution and desired internalization, potentially improving the bioavailability of CBD. Adequate stiffness is a prerequisite for microneedles to penetrate the skin. In our research, the insertion force of the three SD@DMNs surpassed the threshold (0.098 N/needle) required for piercing the stratum corneum [28]. The introduction of combined effervescent ingredients in Ef/CBD-SD@DMNs resulted in stronger mechanical properties compared to NaHCO_3_/CBD-SD@DMNs, leading to deeper skin penetration. In addition, the effervescent components significantly promoted transdermal delivery in both physiological and acidic environments. This is probably because the effervescent components rapidly generate CO_2_ bubbles and actively push the drug deep into the skin. In general, the improved transdermal delivery of Ef/CBD-SD@DMNs can be attributed to the stronger array rigidity and rapid gas generation, which can effectively “blow” the CBD into the deeper layers of the skin.

As described in the introduction section, the downregulation of TRPV expression can inhibit the development of melanoma by increasing the Ca^2+^ influx. In vitro studies have demonstrated that CBD-SD induces B16-F10 cell apoptosis via the “TRPV1-NFATc1-ATF3” pathway. Specifically, increased calcium influx triggered the nuclear translocation of NFATc1, which subsequently downregulated the transcription factor ATF3, leading to melanoma apoptosis. These findings provide insights into the molecular mechanisms underlying the anti-melanoma effect of CBD-SD. Furthermore, sufficient calcium ions are of great importance for CBD to initiate calcium influx, the incorporation of CaCO_3_ provides sufficient calcium for Ca^2+^ influx, underscoring the importance of our Ef/CBD-SD@DMNs design from the aspect of combinational melanoma therapy.

In vivo, the anti-melanoma efficacy of Ef/CBD-SD@DMNs was found to be the most significant among all the treatment groups. Our research confirmed that intra-tumoral CBD increases Ca^2+^ influx, transfers NFATc1 into the nucleus, and then downregulates ATF3 expression, ultimately inducing melanoma cell apoptosis, which validate our aforementioned hypothesis regarding the anti-melanoma efficacy and in vivo mechanisms. Successful intervention in this pathway depends on transdermal delivery and tumoral penetration of CBD. The rationale for incorporating CaCO_3_ into Ef/CBD-SD@DMNs was further verified from both the perspectives of anti-melanoma efficacy and transdermal drug delivery.

In a previous report, it was suggested that glycolytic metabolism within the tumor microenvironment can result in the overproduction of lactic acid and decrease in intra-tumoral pH. This creates a hypoxic environment and disrupts the energy metabolism balance, ultimately leading to the suppression of the TME [44]. The results of this study suggest that Ef/CBD-SD@DMNs can promote M1 repolarization of TAMs and reduce the abundance of immunosuppressive cells, such as M2 TAMs and regulatory T cells, ultimately leading to the activation of the entire TME. This response could be attributed to the elimination of H^+^ within the tumor via effervescence and the introduction of exogenous calcium, which positively modulates the TME towards an anti-tumoral response. However, there are several issues that need to be clarified in this study. Firstly, the specific molecular mechanisms underlying the clearing of the intra-tumoral proton-regulated microenvironment remain unclear. So far, no direct evidence has yet confirmed whether the polarization of M1 TAMs or enhanced calcium ions influx leads to the activation of the TME. Secondly, the direction of gas production in this study is random and lacks sufficient targeting ability, although it can enhance transdermal drug delivery. Additionally, the biodistribution of Ef/CBD-SD@DMNs following transdermal administration remains undetermined. Thus, it is currently unknown how much CBD directly enters the tumor via transdermal delivery and how much is distributed via the blood circulation. These issues will be addressed in future studies to further improve the understanding of the mechanisms underlying Ef/CBD-SD@DMNs action.

## Conclusion

In summary, the novel Ef/CBD-SD@DMNs system developed in this study offers a promising approach to improve the efficacy of CBD-based therapy for melanoma. Ef/CBD-SD@DMNs combines the advantages of effervescence and CBD-based solid dispersion to achieve better transdermal and tumoral delivery of CBD. The in vitro and in vivo results demonstrate that Ef/CBD-SD@DMNs can not only effectively induce melanoma apoptosis via the “Ca^2+^ influx-NFATc1-ATF3” pathway but also activate the tumor microenvironment probably through increasing intra-tumoral pH environment. This study provides a facile and efficient design for a transdermal delivery system that may have a significant impact on the development of new melanoma therapies.

## Electronic supplementary material

Below is the link to the electronic supplementary material.

Supplementary Material 1

Supplementary Material 2

Supplementary Material 3

## Data Availability

All data presented in this paper are included in the main text and the supplementary information.

## Abbreviations

CBD: Cannabidiol
DMNs: Dissolving microneedles
CBD-SD: CBD-based solid dispersion
CBD-SD@DMNs: Cannabidiol solid dispersion-doped dissolving microneedles
NaHCO_3_/CBD-SD@DMNs: Cannabidiol solid dispersion-doped dissolving microneedles with NaHCO_3_
Ef/CBD-SD@DMNs: Effervescent cannabidiol solid dispersion-doped dissolving microneedles
TRPV1: Transient receptor potential vanilloid 1
NFATc1: Nuclear factor of activated T-Cells, cytoplasmic 1
ATF3: Activating transcription factor 3
TME: Tumor microenvironment
TAMs: Tumor-associated macrophages
PVP: Polyvinyl pyrrolidone
SEM: Scanning electron microscope
EDS: Energy dispersive X-ray spectroscopy
XRD: X-ray diffraction
CLSM: Confocal laser scanning microscopy
C6: Coumarin 6
DAPI: 4’,6-diamidino-2-phenylindole
