# Supplementary material for: Effervescent cannabidiol solid dispersion-doped dissolving microneedles for boosted melanoma therapy via the “TRPV1-NFATc1-ATF3” pathway and tumor microenvironment engineering
Source: Biomater Res. 2023 May 18;27:48. doi: 10.1186/s40824-023-00390-x (PMC10193696; doi:10.1186/s40824-023-00390-x)
Supplement: Supplementary file 1 — Supplementary Material 1 [file 40824_2023_390_MOESM1_ESM.docx]

**Supporting Information**

**Effervescent cannabidiol solid dispersion-doped dissolving microneedles for boosted melanoma therapy via** **the “TRPV1-NFATc1-ATF3” pathway and tumor microenvironment** **engineering**

Jiachen Shi^1†^, Qiuling Ma^1†^, Wenting Su^1^, Congyan Liu^1,2^, Huangqin Zhang^1,2^, Yuping Liu^1,2^, Xiaoqi Li^1,2^, Xi Jiang^1^, Chang Ge^1^, Fei Kong^1^, Yan Chen ^1,2^, Ding Qu^1,2,*^

^1^ Affiliated Hospital of Integrated Traditional Chinese and Western Medicine, Nanjing University of Chinese Medicine, Nanjing, 210028, China.

^2^ Jiangsu Province Academy of Traditional Chinese Medicine, Nanjing, 210028, China.

*Corresponding author: Ding Qu, Ph.D., Professor, Jiangsu Province Academy of Chinese Medicine, 100 Shizi Road, Nanjing 210028, China. E-mail: quding1985@hotmail.com

^*^Correspondence to: Ding Qu (E-mail: quding1985@hotmail.com)

^†^ These authors contributed equally to this work.

Full list of author information is available at the end of the article.

**
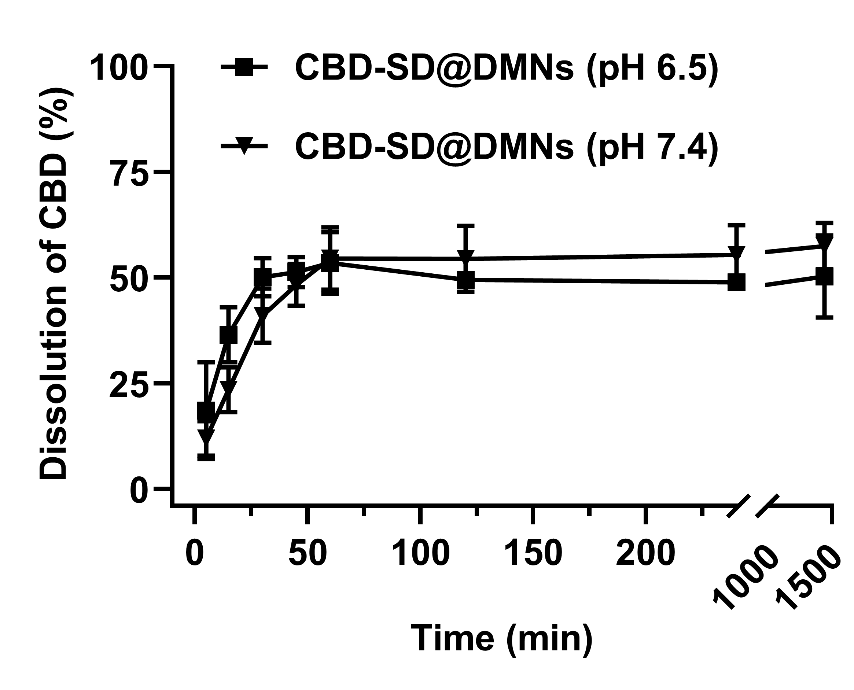
**

**Figure S1.** Cumulative dissolution of CBD from CBD-SD@DMNs at different pH values. Data are represented as mean ± SD, n = 6.


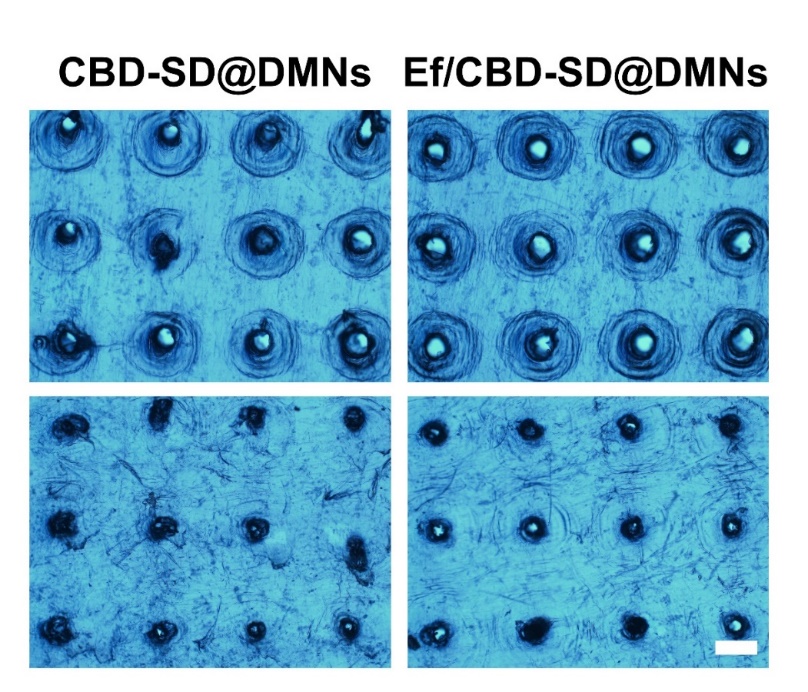


**Figure S2.** The front (top) and back (bottom) of three-layer pierced parafilm of Ef/CBD-SD@DMNs and CBD-SD@DMNs. The bar is 400 μm.


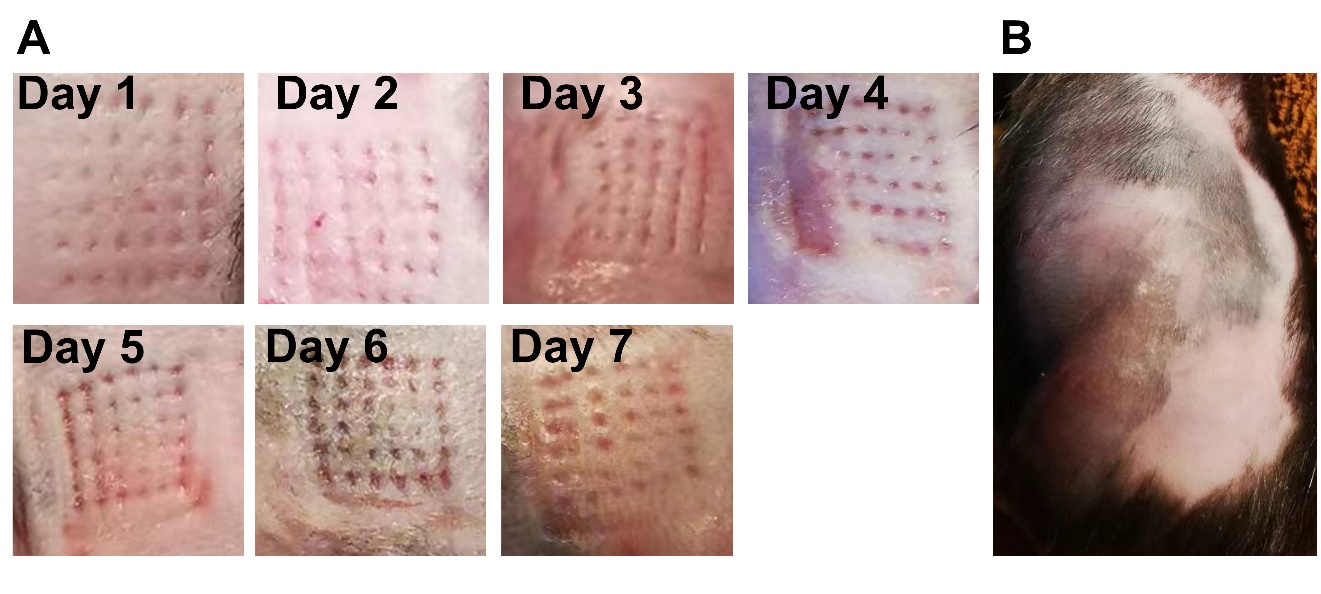


**Figure S3.** (**A**) The image of mouse skin after Ef/CBD-SD@DMNs inserted from day one to seven. (**B**) The image of mouse skin after 10 min of puncture on the seventh day.


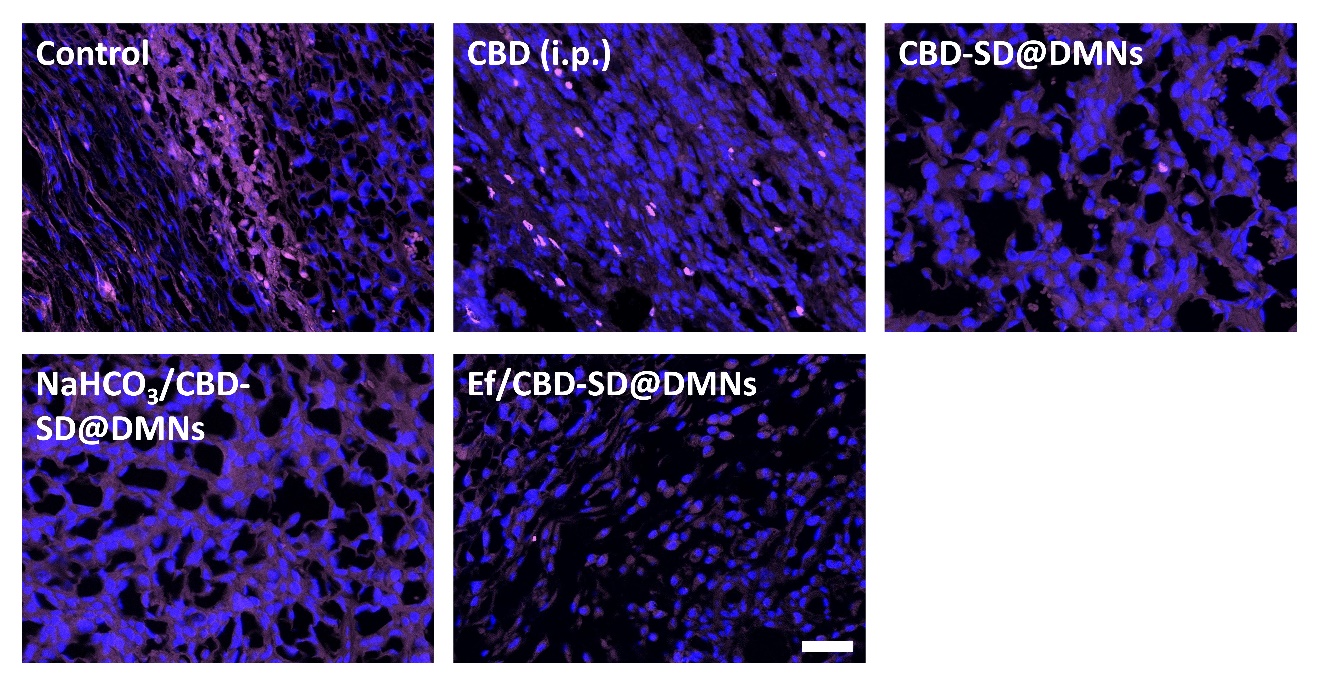


**Figure S4.** Representative immunofluorescence images of tumours showing Foxp3^+^ regulatory T cell for each experimental group. The bar is 40 μm.


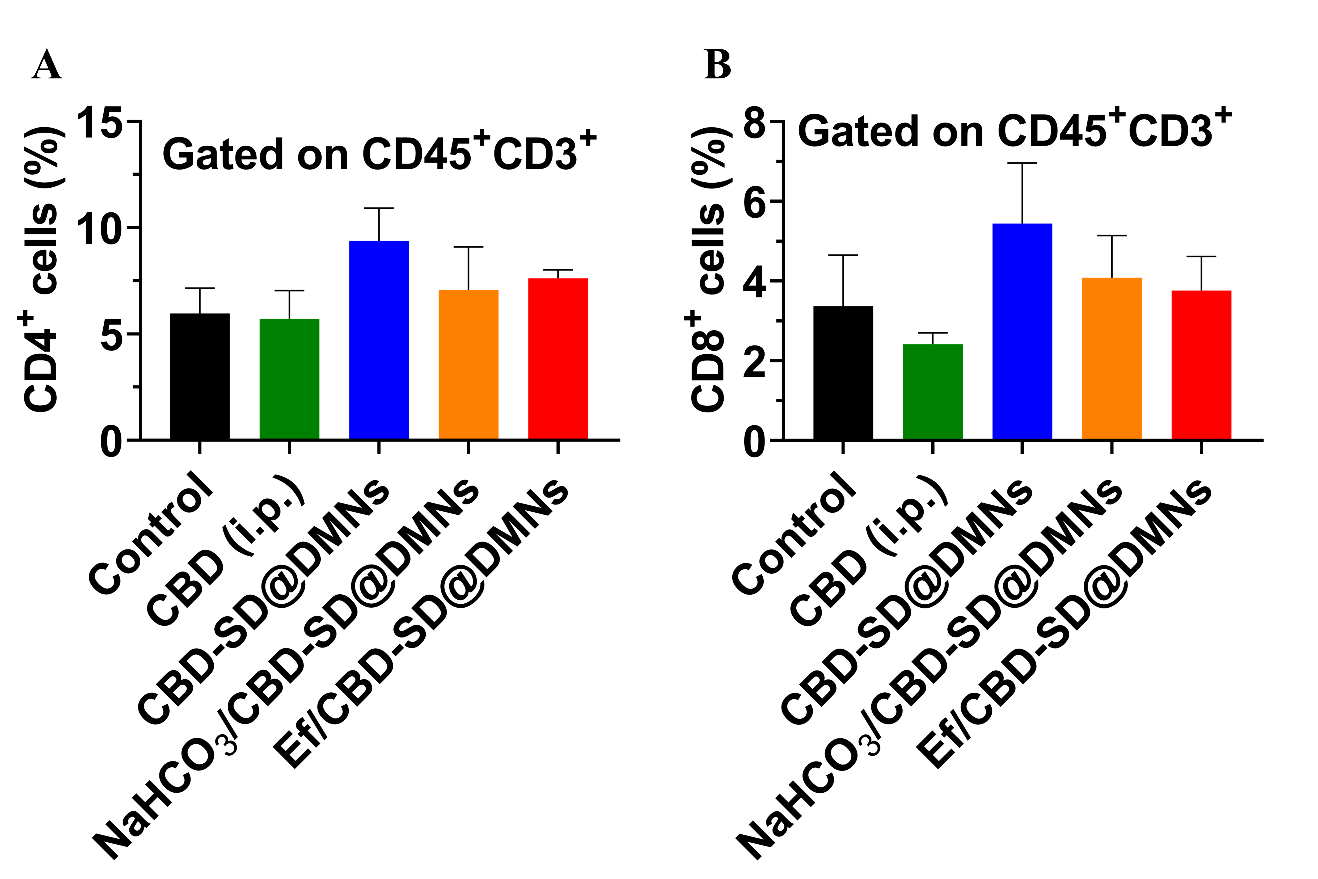


**Figure S5.** Flow cytometric quantification of (**A**) helper T cells and (**B**) killer T cells in spleen tissue from each experimental group. (n = 3,x ± s)
