# Supplementary material for: Effervescent cannabidiol solid dispersion-doped dissolving microneedles for boosted melanoma therapy via the “TRPV1-NFATc1-ATF3” pathway and tumor microenvironment engineering
Source: Biomater Res. 2023 May 18;27:48. doi: 10.1186/s40824-023-00390-x (PMC10193696; doi:10.1186/s40824-023-00390-x)
Supplement: Supplementary file 2 — Supplementary Material 2 [file 40824_2023_390_MOESM2_ESM.docx]

02/02/2023

Prof. Byeongmoon Jeong Editor-in-Chief, Biomaterials Research

Dear Editors,

On behalf of all authors, I am submitting the attached manuscript, “Effervescent cannabidiol solid dispersion-doped dissolving microneedles for boosted melanoma therapy via the “TRPV1-NFATc1-ATF3” pathway and tumor microenvironment engineering” to be considered for publication in ***Biomaterials Research****.*

**Highlight:** The efficacy of dissolving microneedles (DMNs) in treating melanoma is closely tied to their transdermal delivery. **Conventional DMNs face significant challenges in anti-melanoma therapy due to the lack of active thrust to achieve efficient transdermal drug delivery and intra-tumoral penetration.** As shown in the graph abstract below, the effervescent cannabidiol solid dispersion-doped dissolving microneedles (Ef/CBD-SD@DMNs) composed of the combined effervescent components (CaCO_3_ & NaHCO_3_) and CBD-based solid dispersion (CBD-SD) were facilely fabricated by the “one-step micro-molding” method for boosted transdermal and tumoral delivery of cannabidiol (CBD). Upon pressing into the skin, Ef/CBD-SD@DMNs rapidly produce CO_2_ bubbles through proton elimination, significantly enhancing the skin permeation and tumoral penetration of CBD. Once reaching the tumors, Ef/CBD-SD@DMNs on one hand can activate transient receptor potential vanilloid 1 (TRPV1), increasing Ca^2+^ influx and inhibiting the downstream NFATc1-ATF3 signal to induce cell apoptosis; on the other hand, Ef/CBD-SD@DMNs raise intra-tumoral pH environment, triggering the engineering of the tumor microenvironment (TME), including the M1 polarization of tumor-associated macrophages (TAMs) and increase of T cells infiltration. The introduction of Ca^2+^ can not only amplify the effervescent effect but also provide sufficient Ca^2+^ with CBD to potentiate the anti-melanoma efficacy. **Such a “one stone, two birds” strategy combines the advantages of effervescent effects on transdermal delivery and TME regulation, creating favorable therapeutic conditions for CBD to obtain stronger inhibition of melanoma growth *in vitro* and *in vivo*.** This study holds promising potential in the transdermal delivery of CBD for melanoma therapy and also offers a facile tool for transdermal therapies of skin tumors.

**Graph abstract:**


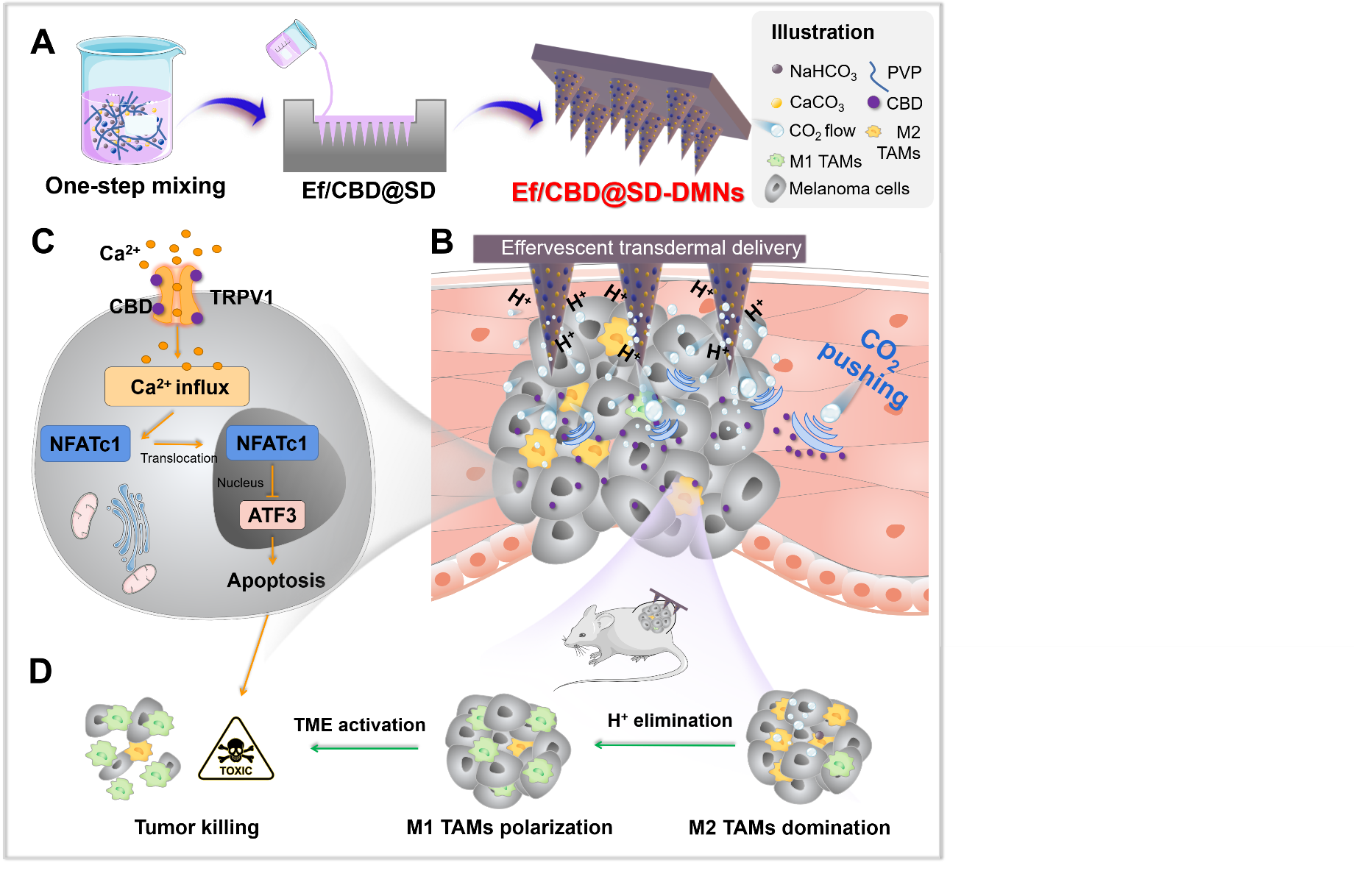


Scheme 1. Schematic illustration of fabrication, active transdermal delivery, and synergistic antitumor mechanism of Ef/CBD-SD@DMNs. The CO_2_ bubbles generated by the combined effervescent components (CaCO_3_ & NaHCO_3_) push CBD deep penetration, consequently improving transdermal and tumoral delivery. The Ca^2+^ influx on one hand triggers the “TRPV1-NFATc1-ATF3” pathway for melanoma apoptosis and on the other hand results in the engineering of the tumor microenvironment, collaboratively boosting anti-melanoma therapy.

This study focuses on synergistic and efficient transdermal drug delivery to boost anti-melanoma efficacy through rational drug combination and well-designed formulations. We believe that our study is likely to be of interest to the readers of ***Biomaterials Research***.

This manuscript has not been published or presented elsewhere in part or in entirety and is not under consideration by another journal. The study design was approved by the appropriate ethics review board. We have read and understood your journal’s policies, and we believe that neither the manuscript nor the study violates any of these. There are no conflicts of interest to declare.

Thank you for your consideration. I look forward to hearing from you.

Sincerely,

Dr. Prof. Ding Qu

Affiliation: Affiliated Hospital of Integrated Traditional Chinese and Western Medicine, Nanjing University of Chinese Medicine, Nanjing 210028, P.R. China; Jiangsu
